# Modern Imaging Technologies of Mast Cells for Biology and Medicine (Review)

**DOI:** 10.17691/stm2021.13.4.10

**Published:** 2021-08-28

**Authors:** I.P. Grigorev, D.E. Korzhevskii

**Affiliations:** Senior Researcher, Laboratory of Functional Morphology of the Central and Peripheral Nervous System, Department of General and Specific Morphology; Institute of Experimental Medicine, 12 Akademika Pavlova St., Saint Petersburg, 197376, Russia; Professor of the Russian Academy of Sciences, Head of the Laboratory of Functional Morphology of the Central and Peripheral Nervous System, Department of General and Specific Morphology; Institute of Experimental Medicine, 12 Akademika Pavlova St., Saint Petersburg, 197376, Russia

**Keywords:** mast cells, histochemistry, immunohistochemistry, toluidine blue, safranin, alcian blue, tryptase, chymase

## Abstract

Mast cells play an important role in the body defense against allergens, pathogens, and parasites by participating in inflammation development. However, there is evidence for their contributing to the pathogenesis of a number of atopic, autoimmune, as well as cardiovascular, oncologic, neurologic, and other diseases (allergy, asthma, eczema, rhinitis, anaphylaxis, mastocytosis, multiple sclerosis, rheumatoid arthritis, inflammatory gastrointestinal and pulmonary diseases, migraine, etc.). The diagnosis of many diseases and the study of mast cell functions in health and disease require their identification; so, the knowledge on adequate imaging techniques for mast cells in humans and different species of animals is of particular importance.

The present review summarizes the data on major methods of mast cell imaging: enzyme histochemistry, immunohistochemistry, as well as histochemistry using histological stains. The main histological stains bind to heparin and other acidic mucopolysaccharides contained in mast cells and stain them metachromatically. Among these are toluidine blue, methylene blue (including that contained in May-Grünwald–Giemsa stain), thionin, pinacyanol, and others. Safranin and fluorescent dyes: berberine and avidin — also bind to heparin. Longer staining with histological dyes or alcian blue staining is needed to label mucosal and immature mast cells.

Advanced techniques — enzyme histochemistry and especially immunohistochemistry — enable to detect mast cells high-selectively using a reaction to tryptases and chymases (specific proteases of these cells). In the immunohistochemical study of tryptases and chymases, species-specific differences in the distribution of the proteases in mast cells of humans and animals should be taken into account for their adequate detection. The immunohistochemical reaction to immunoglobulin E receptor (FcεRI) and c-kit receptor is not specific to mast cells, although the latter is important to demonstrate their proliferation in normal and malignant growth.

Correct fixation of biological material is also discussed in the review as it is of great significance for histochemical and immunohistochemical mast cell detection.

Fluorescent methods of immunohistochemistry and a multimarker analysis in combination with confocal microscopy are reported to be new technological approaches currently used to study various mast cell populations.

## Introduction

Mast cells (tissue basophils, mastocytes, labrocytes) originate from hematopoietic stem cells and occur in nearly all organs and tissues. Their primary function is to maintain homeostasis of organs under environmental conditions. They produce and accumulate a large number of mediators, which are released when mast cells are activating causing different responses in the surrounding tissue. In particular, when responding to entering harmful agents (allergens, pathogens, parasites), mast cells participate in initiating an inflammatory response and in post-traumatic regeneration processes [1–3]. Currently, for some unknown reasons, mast cells can cause an uncontrolled inflammatory response, which results in atopic diseases, such as bronchial asthma, eczema, and allergic rhinitis [1, 4, 5]. Mast cell proliferation and acceleration are the main features of such human disease as mastocytosis [6]. Mast cells accumulating in the synovial tissue in rheumatoid arthritis indicate their possible involvement in this chronic inflammatory disease [7]. Mastocytes accumulate in atherosclerotic plaques and, probably, play a critical role in developing infarction and stroke [4, 8, 9]. Mast cells have been shown to participate in the pathogenesis of endometriosis, Crohn’s disease, and inflammatory bowel disease, as well as in the development of pulmonary and hepatic fibrosis [8, 10, 11]. Some of the mediators mast cells secrete can contribute to tumor growth-stimulating angiogenesis and malignancies, while others can cause the apoptosis of tumor cells [9, 12]. Recently, there has been extensively debated a question of mast cell contribution to the pathogenesis of a number of nervous diseases and psychiatric disorders (multiple sclerosis, migraine, amyotrophic lateral sclerosis, autism spectrum disorders, depressions, and others) [8, 13–15].

Since mastocytes provide essential functions and participate in the pathogenesis of many diseases, a great deal of research works, both clinical and carried out on experimental models of diseases, are devoted to mastocytes. Many findings concerning mast cell detection in human and animal tissues seem to be controversial due to the methodological restrictions and the interpretation peculiarities of cytological and histological data; in particular, when comparing the results obtained on different species of animals and on humans. Therefore, the knowledge on adequate imaging techniques for mast cells in humans and different species of animals under different conditions is of particular importance.

On this basis, **the aim of the present study** was to analyze the technological characteristics of the techniques used to identify human and animal mast cells for correct selection of informative methods when conducting research and diagnostic studies.

## Morphological and biochemical characteristics of mast cells

Mastocytes do not possess the form and localization specific of only this type of cells, therefore, they are indirectly imaged on histological preparations, by detecting the substances specific to these cells using histochemistry and immunohistochemistry. In the cytoplasm of mast cells, there is a variety (over two hundred) of compounds including biogenic amines (histamine, serotonin, dopamine), proteoglycans (serglycin), mucopolysaccharides (heparin, chondroitin sulfate), proteases (tryptases, chymases, carboxypeptidase А), cytokines, growth factors, hormones, and other bioactive compounds [1, 10, 16–18].

Mast cells in certain organs significantly differ by the composition of mediators [19]. The difference in dye affinity of mast cell granules in different organs gave grounds to L. Enerbäck as early as in 1966 to distinguish these cells in rodents into two types: mucosal cells and connective tissue, or serosal, cells [20, 21]. Comparison of distribution and mediator composition of rodent and human mast cells enables to apply the classification to mastocytes of other animals and human.

The composition of mediators contained in the granules of mastocytes of different types is different. In connective tissue cells, there is much heparin, while mucosal cells hardly contain it; however, in mucosal cells, there are many other acidic mucopolysaccharides — chondroitin sulfate А and dermatan sulfate (previously referred to as chondroitin sulfate В), which have not been found in connective tissue mastocytes [1, 22]. It should be noted that heparin in human body was found only in mast cells, it accounting for about 75% of total mucopolysaccharides of mast cells, and chondroitin sulfates make up their remaining part [23, 24].

## Histochemical detection of mast cells

The plenty of sulfated glycosaminoglycans, primarily, heparin, in secretory granules, and their localization in mast cells enable to image selectively these cells using histochemical staining. Negatively charged sulfate groups of glycosaminoglycans interact with main aniline dyes, e.g., toluidine blue, methylene blue, or thionin. A dye molecule covalently bound to sulfated glycosaminoglycans changes its color, and mast cells are stained metachromatically, i.e. different from orthochromatic color of the dye itself [25]. Histochemical method of metachromatic staining is widely used to detect mastocytes on histological specimens, since it is relatively simple, low-cost, efficient, and is applicable to nearly any human and animal tissues. However, the detectability of mastocytes significantly depends on the array of factors: a type and maturity of mast cells, a test tissue type, animal species, a used dye, incubation solution pH and the staining duration, fixation solution type, fixation time, and the final processing technique of stained preparations [21, 26–33]. More frequently, for mast cell detection, there is used metachromatic staining by toluidine blue or Romanowsky stain (May-Grünwald– Giemsa staining), as well as a combined staining by alcian blue and safranin.

In ***toluidine blue*** staining mast cells exhibit intensive dark violet metachromasy in contrast to other type cells, which are blue stained (orthochromatically). Widespread application of toluidine blue has resulted in the invention of several stain variants and enabled to reveal a number of characteristics and limitations of the technique. Toluidine blue for metachromatic detection of mast cells is used in three main varieties: 1) 0.5% neutral water solution; 2) 0.5% acidic solution (pH 0.3–4.0); 3) 0.5% acidic solution in 70º ethanol (the so-called toluidine blue according to Luna protocol [34]). For more effective staining of mast cells, it is more preferable to use an acidic solution of toluidine blue with pH 0.3–4.0 [21, 35].

It is important to keep in mind that heparin, toluidine blue binds with, is deficient in mucosal mastocytes, and is abundant in mature connective tissue mastocytes; in immature cells, its content is low and grows as far as cells differentiate [36, 37]. As a result, mature connective tissue mast cells are stained metachromatically by toluidine blue more intensively and fast, while mucosal cells and immature connective tissue mastocytes require a long-term staining — up to 2 and even 5 days [30, 38, 39]. For accurate calculation of mast cell count and obtaining correct results, staining time should be standardized.

Mast cells staining with toluidine blue depends on a fixation technique. After 10% formalin fixation, toluidine blue stains well connective tissue mast cells in different organs in human and animals, although mucosal mastocytes are stained poorly or fail to stain [30, 32, 40, 41]. To improve mucosal mastocyte staining, it has been suggested to wash fixed in formalin specimens in tert-butanol [42]; however, the best results are shown by using non-aldehyde (non-formalin) fixative. Many studies have demonstrated Carnoy’s fixative to enable to reveal more mast cells (including mucosal ones) in toluidine blue staining than formaldehyde-based fixatives [35, 38, 43, 44]. In addition to Carnoy’s fixative, the fixatives containing metals preserve well metachromasy of mast cells: basic lead acetate, which is also referred to as Mota fixative [20, 35, 45], and zinc-containing fixatives [46–52].

It should be taken into consideration that toluidine blue, apart from mast cells, can cause metachromasy in intracellular granules of macrophages and basophilic leukocytes, as well as in goblet cells of intestine in human and animals [30, 52, 53].

***Alcian blue*** combined with ***safranin О*** (which is usually called simply safranin) is also widely used for mast cell visualization. The advantage of the method is in that it enables to detect simultaneously mast cells of both types: serosal and mucosal cells. Alcian blue stains mucosal mast cells blue, and safranin stains selectively serosal cells in red and pink. It is due to the fact that safranin binds to highly sulfated glycosaminoglycans including heparin, which is typical for connective tissue cells, while alcian blue stains the granules of mast cells containing poorly sulfated glycosaminoglycans, primarily, heparin precursors and chondroitin sulfate Е, which are typical for mucosal mast cells [32, 42, 54]. Therefore, mastocyte granules, which are stained pink- and red-colored by safranin, contain heparin, and those stained blue by alcian blue do not contain heparin.

Moreover, considering heparin distribution in mature and immature mastocytes, they can be differentiated using a complex staining with alcian blue/safranin: mature (connective tissue) mastocytes are pink stained by safranin, while immature (connective tissue and mucosal ones) are blue stained by alcian blue. When maturated, in staining connective tissue mastocytes there is a gradual shift: at an intermediate stage some granules are pink stained, though there is a persisting blue rim caused by alcian blue, or the cytoplasm becomes violet, while both mature and immature mucosal mastocytes preserve their blue color with alcian blue [55–58].

Alcian blue detects more mast cells at low pH: e.g., at pH 1.0 it stains more mastocytes than at pH 3.5. Alcian blue and safranin staining efficiency depends on a fixation method: it is preferable to use non-aldehyde fixative (e.g., Carnoy’s fixative or Mota fixative) [26, 41, 59].

When using alcian blue, one should bear in mind that in the intestine it stains both: mast cells and goblet cells [43, 52], and in the airways — goblet cells and Clara cells [60, 61].

Combined staining by alcian blue and eosin according to Sukhorukova and Voronchikhin enables to detect simultaneously mast cells and eosinophilic leukocytes — basic cell populations participating in bronchial asthma pathogenesis [62].

In some cases, to detect mastocytes in the combination of alcian blue and safranin, the first dye is substituted by ***astra blue*.** Astra blue, like alcian blue, belongs to a group of copper-bearing phthalocyanine dyes, and under certain application conditions it stains mast cells more selectively than some other dyes including toluidine blue; moreover, astra blue gives no background stain [63, 64]. When used in combination with safranin, astra blue, as alcian blue, stains immature and/or mucosal mast cells, while safranin stains heparin-containing mature serosal mastocytes [21, 39]. Similar to other dyes, astra blue stains well mast cell after fixation in Carnoy’s fixative or Mota fixative rather than in formalin [63].

Oftentimes, Romanowsky stain in different variations for histological staining is used to detect mast cells. So, in ***May-Grünwald–Giemsa*** staining the cytoplasm of mast cells gains dark blue color, while the granules are red-stained [45]. The method reveals mucosal mast cells, and sometimes more effectively compared to toluidine blue, alcian blue, astra blue, or enzyme histochemistry or immunohistochemistry used (see below) [53, 65–67]. It should be noted that although May-Grünwald–Giemsa stain used to detect mast cells is highly selective, the procedure takes more time than toluidine blue staining [67].

Most researchers use independently the main component of Romanowsky and May-Grünwald–Giemsa stain — methylene blue and its oxidates, which form in the stain — azure A and azure B (a synonym is azure I). Metachromatic staining of mastocytes using ***methylene blue*** is widespread, it selectively labels mast cells giving less background staining compared to alcian blue [52, 68, 69]. Methylene blue stains more mastocytes after fixation in Carnoy’s fixative or Mota fixative than in formalin [68, 70].

***Azure A*** is mentioned in different manuals as a frequently used dye for mast cells imaging, however, in fact, it is rarely used. Azure A was demonstrated to reveal serosal and mucosal mast cells as well as other dyes (also using enzyme histochemistry); moreover, at lower pH (0.5–1.0) azure A detects more mastocytes than at pH 3.0–4.0 [20, 71]. It is of interest that at pH 0.5 azure A stains mast cells orthochromatically, and at pH increased up to 4.0 there is a shift to metachromasy [20]. There is evidence that azure A in contrast to other dyes fails to stain mastocytes in rat brain [72].

More rarely, for metachromatic staining of mastocytes, other dyes are used: ***cresyl violet*** [73] or ***thionin.*** Thionin ranks below in mast cell detection efficiency compared to toluidine blue and gives more background staining [72, 74, 75]. There is evidence for effective mast cell staining with ***brilliant green*** [76], ***methyl green*** [77], and ***methylene green*** [78].

In 1958, the Russian histologist M.G. Shubich suggested using 0.5% acidic solution of ***basic brown*** (a synonym is Bismarck brown) for selective mast cell staining [79]. The dye combines with acidic mucopolysaccharides and contrastively stains the granules of mast cells in yellow-brown without staining the nucleus and other type cells. Specimens are well stained regardless of a fixative used (absolute alcohol, alcoholic formalin, 4% lead acetate, a mixture of lead acetate and formalin, etc.). The technique is simple and selectively detects mastocytes just as well as toluidine blue [80, 81].

Carbocyanine dye, pinacyanol, combined with erythrosin ***(inacyanol–erythrosinate)*** is known as a selective dye for mast cells, which is used in histological practice for metachromatic staining of human and animal mastocytes. In the material fixed in formalin, pinacyanol–erythrosinate detects more mastocytes than toluidine blue [82, 83]. However, the stain is worse than toluidine blue, since it stains the cytoplasm only, and fails to image the granules clearly [82]. Pinacyanol disadvantage is its high sensitivity to heat and light; due to that stained preparations quickly discolor.

There has been suggested a method for staining mast cells using an acidity change indicator — ***ferroin.*** The method is easy-to-use and detects mast cells not less effectively than toluidine blue or alcian blue– safranin [84].

The major histological staining method — ***hematoxylin and eosin*** staining — is ***non-effective*** for selective mast cell staining [85]. Hematoxylin has no affinity for mast cell granule components; therefore, it is frequently used to counterstain cellular nuclei in immunohistochemical staining of mastocytes.

In addition to classical histological dyes for visualization of connective tissue mast cells, fluorescent dyes are used, chiefly, ***berberine sulphate.*** Berberine is a cationic dye, which exhibits high affinity for heparin and poor proper fluorescence, although after binding to heparin it emits intense yellow fluorescence [30, 44, 86]. Berberine intense fluorescence is directly proportional to heparin amount that enables to use it both for labeling mature connective tissue mastocytes containing heparin on histological preparations [55, 56] and also for cytofluorometrical measurements of heparin amount in mast cells [54]. Cytoplasmic granules of mastocytes fluoresce under berberine more contrastively compared to the background tissue at low pH of the dye (1.2–2.0). Berberine is used to label mastocytes both in fluorescent microscopy, and also in light microscopy: it stains mast cells in red-brown color [87]. Since berberine labels only serosal (heparin-containing) mast cells, it is used in the combination with alcian blue for differential staining of serosal (by berberine) and mucosal (by alcian blue) mast cells [21], or together with toluidine blue that enables to distinguish serosal berberine-labeled mastocytes from the general population of mastocytes stained by toluidine blue [88, 89].

Berberine staining is sensitive to a fixation method: buffered neutral formalin almost completely suppresses fluorescence; Carnoy’s fixative is recommended for successful detection [41, 90]. It should be taken into consideration that although berberine stains well mouse and rat mastocytes, according to some data [43, 44], it fails to cause fluorescence in connective tissue mast cells of guinea pigs and in human skin. The disadvantage of the stain is fragility due to short-term fluorescence of berberine.

In addition to berberine, ***avidin*** glycoprotein selectively binds to heparin contained in the granules of connective tissue mast cells [91]. Owing to this property, avidin conjugated to horseradish peroxidase, fluorescein isothiocyanate (FITC), rhodamine, or other label, is used to detect connective tissue mast cells under light or fluorescent microscopy. Selectability of avidin binding to heparin is proved by the usage of heparanase — an enzyme, which destroys heparin and completely prevents staining with avidin [92]. Avidin binds to heparin both: selectively and quantitatively, i.e., fluorescence intensity of avidin conjugated to fluorescein is proportional to heparin amount in mastocyte granules, and it is used for measuring the amount of heparin and heparin-containing granules in mast cells [92]. By means of avidin binding to heparin, it is possible to observe a degranulation process — the release of secretory granules from mast cells [86, 93, 94].

Due to high affinity for heparin, avidin detects connective tissue mast cells not less effectively than toluidine blue, and sometimes even more effectively [38, 44, 71]. It surpasses berberine since it was not found to have species-specific limitations for mastocyte staining: avidin enables to label serosal mast cells derived from mice and rats, and also, in contrast to berberine, from human and guinea pigs [44, 95]. It is important to keep in mind that avidin affinity for heparin is so high that it can bind to heparin even forming a part of an avidin–biotin-peroxidase complex, which is used in immunohistochemistry, not related to mast cell study resulted in nonspecific staining of mast cells. There have been developed specific methods to prevent an artifact [96]. Mastocytes imaged by avidin show better findings after fixation in Carnoy’s fixative compared to a formalin-containing fixative [44].

Occasionally, ***acridine orange*** is used for mast cell imaging; as it binds with mast cell granules, the latter starting to emit orange-yellow fluorescence. Moreover, it stains mastocyte granules in orange color in the transmitted light that enables to see labeled objects on histological preparations under a light-optical microscope. There is evidence that acridine orange can form bonds with heparin, and it is likely to stain serosal but not mucosal mastocytes [41]. The capability of acridine orange to combine with cytoplasmic granules is used to study degranulation processes in health and in apoptotic destroy of mastocytes [97].

It should be remembered that acridine orange fluorescence is short-time. However, its main application problem is that as a cationic dye it can bind to different acidic vesicles, lysosomes, and nucleic acids [98]. As a result, acridine orange induces fluorescence of nucleic acids in the nucleus, and stains vesicles and lysosomes in cells of various nature [98, 99], it is the evidence for low specificity of the dye in relation to mast cells.

Above described histochemical stain types for mast cells are based on the binding of a basic dye to heparin and other acidic mucopolysaccharides; however, heparin, although in less amounts, can be found in basophilic leukocytes (basophils), which are also stained by basic dyes. To overcome the disadvantage, there have been developed more specific enzyme histochemical and immunohistochemical techniques to identify mastocytes; the techniques being based on detecting the enzymes — ***tryptase*** and ***chymase*** — specific for mastocytes. These are serine proteinases, which exhibit, respectively, trypsin-like or chymotrypsin-like characteristics, and amount up to 35–50% of total proteins of mastocytes [33, 100]. Tryptase and chymase were found in basophils, but in concentrations by two orders lower than in mastocytes [101]. Therefore, when using immunohistochemistry with signal amplification sufficient to reveal mast cells, basophilic granulocytes are not detected.

According to the presence of certain serine proteinases, mast cells are divided into those containing tryptase, chymase, or both enzymes. Human connective tissue mast cells produce and store in secretory granules tryptase and chymase, while mucosal cells — only tryptase (Table 1) [100, 102].

**Table 1:** Serine proteinase of human mast cells and laboratory animals

Species	Serosal mast cells		Mucosal mast cells	
	Tryptases	Chymases	Tryptases	Chymases
Human	Tryptase	Chymase	Tryptase	None
Mouse	mMCP-6, mMCP-7	mMCP-4, mMCP-5	None	mMCP-1, mMCP-2
Rat	rMCP-6, rMCP-7	rMCP-1, rMCP-5	None	rMCP-2, -3, -4, -8, -9, -10

Mast cells of laboratory rodents significantly differ in the composition of proteases from those in humans: human mucosal mastocytes have tryptase only, while rodents have only chymase; serosal mast cells contain both: tryptase and chymase — in human and in rodents. In mice, serosal mast cells synthesize and store two chymases in granules — mouse mast cell protease-4 and -5 (mMCP-4 and mMCP-5) and two tryptases — mMCP-6 and mMCP-7, while mucosal mast cells produce only chymases; moreover, they are different from those contained in connective tissue mastocytes (mMCP-1 and mMCP-2), but do not synthesize tryptases [100, 102]. In rats, serosal mast cells also contain tryptase and chymase: two types of tryptase — rat mast cell proteases: rMCP-6 and rMCP-7 — and two chymases — rMCP-1 and rMCP-5, while mucosal mastocytes express several chymases (rat mast cell proteases: rMCP-2, -3, -4, -8, -9, -10), but have no tryptases [18, 103].

Species-specific proteases were found in guinea pigs, rabbits, Mongolian gerbils, golden hamsters, cats, dogs, and other animals [104, 105], and a distribution pattern of chymases and proteases in serosal and mucosal mast cells in different animals is different. This fact determines the impossibility of interspecies standardization of immunohistological techniques used to detect mast cells, when their cytospecific enzymes are labeled.

## Enzyme histochemistry

An enzyme histochemical technique is useful for labeling specific mast cell enzymes. In the middle of the ХХ century mast cells were found to have specific for them esterase (chloroacetate esterase) activity, its localization can be defined using Leder’s enzyme histochemical reaction. For the technique the following components are used: naphthol-AS-D chloroacetate as a specific substrate and freshly prepared diazonium dye: fast garnet GBC, fast blue BB, fast red violet LB, pararosaniline, or basic fuchsin. Enzyme hydrolysis of ester groups by mast cell esterase releases from a substrate the naphthol, which combines with diazonium dye-forming highly stained deposits in the enzyme activity site, thereby labeling mast cells [85]. The advantage of the method is in the fact that compared to other enzyme histochemical tests, this one can be successfully used on paraffin tissue sections [85].

As well as other histochemical reactions, the chloroacetate esterase reaction depends on a fixation type of the test tissue; however, the data on this topic are conflicting: different researchers carrying out Leder’s reaction after fixation in formalin, Carnoy’s fixative or Mota solution obtained radically different results [42, 70, 106].

Chloracetate esterase-labeled cells are also stained metachromatically by toluidine blue, Giemsa stain, or alcian blue suggesting their mast cell nature [42, 106, 107]. However, along with mastocytes, chloroacetate esterase reaction (as a rule, with low intensity) is found in polymorphonuclear leukocytes [106].

There is evidence suggesting that naphthol-AS-D chloroacetate esterase reaction is the representative of chymase activity, and therefore, the localization of the enzyme histochemical reaction products corresponds to chymase localization sites in mastocytes [69, 106]. On this basis, and considering chymase distribution in mast cells of different types (see Table 1), it can be believed that chloroacetate esterase reaction enables to reveal in human serosal mast cells, while in mice and rats — both: serosal and mucosal mastocytes.

Some other chymotryptic proteases, e.g., elastase and cathepsin G, exhibit substrate specificity of chymase, moreover, they can be found both: in mastocytes and other types of human and animal cells, therefore, chloroacetate esterase reaction can detect more cells than there are chymase-containing mastocytes in the region of interest [29, 69, 108]. To overcome the disadvantage there were developed substrates (peptide compounds) that are more selective for chymase, as well as the substrates specific for tryptase that enabled to carry out an enzyme histochemical reaction to detect the localization of chymase or tryptase with high selectivity [38, 41, 109]. The specificity of an enzyme histochemical reaction to chymase and tryptase can be checked using different protease inhibitors: e.g., histochemical reaction to chimase is blocked by chymostatin, a soybean trypsin inhibitor and phenylmethylsulfonyl fluoride, while that to tryptase — by leupeptin and some other oligopeptides [106, 110].

It should be noted that using this reaction we can image active enzymes able to cleave specific substrates. However, there is evidence that a part of chymase and tryptase in mastocytes is inactive, and therefore, they cannot be detected by an enzyme histochemical reaction [108, 109]. In this case, inactive, as well as active enzymes, can be revealed by immunohistochemistry.

## Immunohistochemistry

Immunohistochemistry is certainly the most sensitive and selective, although costly, labor-intensive, and highly technical method to identify mast cells. As other techniques, this one detects the compounds selectively typical of mast cells including, primarily, tryptase, and chymase. As previously mentioned, these enzymes exhibit species specificity; therefore, the antibodies to them are also specific for each species. Conjugated to a corresponding label, the antibodies can be revealed on preparations using light, fluorescent, and electron microscopy.

It is immunohistochemistry that showed human mast cells to be divided into two groups: tryptase-immunoreactive (MC_T_) and tryptase- and chymase-immunoreactive (MC_TC_) MC_T_ distribution in body tissues corresponds to the distribution of mucosal mastocytes (primarily, in the lungs and gastrointestinal mucosa), and MC_TC_ — to that of connective tissue mast cells (in skin, gastrointestinal submucosa, peritoneal fluid) [95, 101, 111]. Thus, immunohistochemical detection of tryptase enables to reveal the entire population of mast cells in human tissues, while supportive chymase immunostaining facilitates to identify a mast cell subtype. In addition to two specified subtypes, some human mast cells containing chymase alone (without tryptase) were revealed by immunohistochemistry (as well as enzyme histochemistry): MC_C_ were described in placenta, the lungs, gastrointestinal mucosa and submucosa, the kidneys, skin, and conjunctiva [95, 112–115]. However, a question of existing in human body the subpopulation of chymase-immunopositive/tryptase-immunonegative mastocytes remains debating, since some researchers received contrary results [116, 117]. It is not improbable that the detection of chymase-immunopositive/ tryptase-immunonegative mast cells is related to low tryptase expression in some mastocytes and/or the complexity of tryptase (as well as chymase) detection by immunohistochemistry using double labeling when correct incubation time with a corresponding antibody is of primary importance [31, 118].

Currently, there are commercially available species-specific antibodies to some mouse and rat chymases and tryptases, which are used in immunohistochemical studies as markers of a certain subgroup of mast cells [87, 108, 119, 120]. Antibodies to tryptase and chymase of human, mice, or rats, according to manufactures’ recommendations, can be used for immunohistochemical study of other animals’ mastocytes; however, availability of such usage of antibodies requires special testing in any single case. In some cases, genetic data on orthologic proteases is considered — those, which are encoded by an identical gene in two different species of animals. For instance, the antibodies to orthologic rat chymase rMCP-1 were successfully used for immunohistochemical detection of murine chymase mMCP-4 [108].

When using immunohistochemistry to obtain adequate results, a fixation technique of the material is of importance. The comparison of the effect of fixation in formalin, Carnoy’s fixative, ethanol, and other fixatives on immunohistochemical detection of mast cells has brought contradicting results [70, 111]. According to our observations, the use of zinc-containing fixatives provides best results. It should be taken into consideration, when carrying out immunohistochemical reactions to mast cell enzymes, that tryptase and chymase are expressed rather late in differentiating mastocytes; due to this, the antibodies to these proteases cannot be used to identify immature mast cells.

In some cases, to label mast cells there is used immunohistochemistry for ***c-kit*** receptor protein (synonym — CD117) — tyrosine kinase transmembrane receptor, a ligand of which is a stem cell factor. The interaction between the ligand and c-kit receptor was found to regulate migration, maturation, and proliferation of mast cells *in vivo* and *in vitro* [6, 75, 121]. C-kit receptor protein is found not only in growing mastocytes, as might be expected based of the established functions, but also in mature mast cells, therefore, the antibodies to c-kit protein enable to detect immunohistochemically the most population of mast cells. For some tissues and animal species, an immunohistochemical reaction on c-kit was demonstrated to reveal mast cells better than toluidine blue [122], and not less effectively than tryptase immunohistochemistry [123]. However, other studies revealed that this marker is not sufficiently specific, and using antibodies to c-kit it is possible to image only a part of mast cells, which are immunopositive to tryptase or chymase [124, 125], and vice versa, only a part of c-kit-immunoreactive cells were also immunopositive to tryptase or chymase [125]. This is due to the fact that c-kit receptor, in addition to mastocytes, is detected in stem and germinal cells, epithelial cells of sweat glands, ducts of the mammary gland, renal and testicular tubules, interstitial cells of Cajal, melanocytes, and basal cells of the epidermis, some neurons and gliocytes of the brain [126–128]. The differential characteristic of c-kit localization in mast cells is their position on the membrane, while in cells of other nature c-kit is localized in the cytoplasm [129].

Moreover, c-kit receptor protein intensively expresses in tumors of different nature, and its detection using immunohistochemistry is of diagnostic value [126, 127, 130–132]. Thus, c-kit protein can be recognized as an optional marker of mast cells: c-kit- immunopositive cells, with high probability, although not definitely, can be mastocytes.

Some other components typical of mast cells are also used as possible markers of mastocytes. One of them is ***high-affinity receptor to immunoglobulin Е (FcεRI)*,** which generally co-expresses with c-kit in mature mast cells [2, 6]. Most mast cells stained with immunohistochemical reaction for tryptase or chymase are also immunopositive for FcεRI [125, 133]. However, in some body parts, e.g., in the human lungs, only a few mastocytes are FcεRI immunoreactive [133]. Moreover, all the tissues studied were found to have significant amount of FcεRI-immunoreactive cells, which were tryptase- and/or chymase-immunonegative [125], that is not surprising, since FcεRI is found not only in mastocytes but also in basophils, monocytes, eosinophils, and epidermal Langerhans’ cells [134, 135]. Thus, although FcεRI is a typical component of mast cells, it (as well as c-kit) cannot be recognized as a selective marker of mastocytes.

***Histamine*** immunohistochemistry was suggested to detect mast cells in different human and animal organs [87, 136, 137]. However, the method has an essential fault due to the fact that not all mast cells have histamine [137, 138], although there is a significant amount of it in neurons, enteroendocrine cells of the gastrointestinal tract, basophils, neutrophils, monocytes/macrophages and dendritic cells [139, 140]. The data indicate that an immunohistochemical (likewise histochemical) reaction to histamine cannot be considered a reliable and selective technique for mast cell detection.

***Serotonin*** (as well as tryptophan hydroxylase — a key enzyme of serotonin synthesis) is contained in human and animal mast cells. Basic information on this issue was obtained from the studies on animals, while data on serotonin in human mastocytes is few. In rodents, serotonin was found, primarily, in connective tissue mastocytes [33, 141]. Some researchers failed to find serotonin using immunohistochemistry in mast cells of cat and dog skin [138, 142], and in the brain of experimental animals and birds it was revealed only in some mast cells [120, 143, 144]. On the other hand, serotonin can be found (apart from mastocytes) in many other cell types of human and animal organs; among them are enterochromaffin cells of the gastrointestinal tract, which produce the great majority of serotonin in the body; various endocrine cells of the lungs, adrenals, prostate, epididymis [145–148]; serotonergic neurons of the central and peripheral nervous system [149, 150]; type III gustatory receptor cells and glomus cells of carotid body [151, 152]. Serotonin is found in epithelial cells of excretory duct lining of different glands [148], trophoblasts and decidual cells of human placenta [153], keratinocytes, fibroblasts, melanocytes, and, probably, other skin cells [154, 155], synovial fibroblasts [156], platelets, lymphocytes and monocytes [157]. Thus, although serotonin was found in human and animal mast cells, it is not specific for mastocytes and is not their selective marker.

Obtaining specific antibodies to ***heparin*** enabled to carry out immunohistochemical studies of this component of mast cell granules. Using the technique, heparin was truly established to be contained in connective tissue mastocytes, but not in all mastocytes [120, 158]; therefore, heparin immunohistochemistry can be used to detect a mast cell type [158]. However, the method is significantly more labor-intensive and costly than currently accepted histochemical techniques of heparin detection.

Immunohistochemical reaction to ***vascular endothelial growth factor receptor-1 (VEGFR-1)*** was suggested for mast cells visualization [159]. However, the data on the receptor presence on mast cells is few, and its frequency of occurrence on mastocytes is unknown. On the other hand, VEGFR-1 was found to be detected in vascular endotheliocytes, epithelial cells of the uterus and bronchi, type 2 pneumocytes, cytotrophoblast, interstitial cells of testicles, T lymphocytes, monocytes, microglial and astroglial cells, and some others [160–163]. Moreover, VEGFR-1 intensively expresses in tumor cells of different location, and it is suggested to be used for diagnostic and therapeutic purposes [164, 165]. Therefore, there is no need to consider VEGFR-1 selective enough to be used for reliable identification of mast cells.

Among the techniques used for direct imaging of human and animal mast cells, microscopy of stained preparations in transmitted light of visible range is the most common and easy-to-use. Fluorescent microscopy and electron microscope are less common. The most informative technique enabling to receive maximum data on the structure and functional condition of certain mast cells is fluorescent immunohistochemistry combined with multimarker confocal microscopy [154, 166–168].

## Conclusion

Table 2 represents the findings of the present critical analysis of methods and histotechnological techniques used for mast cell imaging in biomedical studies and clinical diagnostics, it shows histo- and immunohistochemical reactions to 10 components typical of mast cells and a limited number of other cell elements to be most frequently used as markers of human mast cells.

**Table 2:** Human mast cell markers and mast cell detection techniques

Markers	Cell type	Staining technique	Additionally stained structures	Notes
1. Sulfated glycosaminoglycans (including heparin)	Mature serosal mastocytes, a part of mucosal mastocytes	**Toluidine blue Methylene blue** Thionin **Safranin O** Basic brown Methylene green Azure A May-Grünwald–Giemsa	Macrophages, basophilic leukocytes, goblet cells, Clara cells	Toluidine blue and methylene blue staining is the easiest and most effective method to detect most mastocytes
2. Heparin	Mature serosal mastocytes	**Berberine Avidin** Acridine orange Immunohistochemistry	Basophilic leukocytes For avidin — any cells containing biotin	Berberine, avidin are highly selective markers of heparin, but berberine does not cause fluorescence in human skin mastocytes
3. Chondroitin sulfate Е	Mucosal and immature mastocytes	**Alcian blue** Astra blue	Components of connective tissue intercellular substance	Frequently used in combination with safranin O for simultaneous detection of mucous and serosal mastocytes
4. Tryptase	Serosal and mucosal mastocytes	Leder’s reaction **Immunohistochemistry**	None	Tryptase immunohistochemistry is the most effective technique to detect human mastocytes Leder’s reaction reveals an active enzyme alone
5. Chymase	Serosal mastocytes	Leder’s reaction **Immunohistochemistry**	None	Leder’s reaction reveals an active enzyme alone
6. c-kit (CD117)	Mastocytes	Immunohistochemistry	Stem cells, germinal cells, epithelial cells, interstitial cells of Cajal, melanocytes and basal skin cells, neurons, gliocytes	Also used as a tumor marker
7. Immunoglobulin receptor Е (FcεRI)	Mastocytes	Immunohistochemistry	Basophilic leukocytes, monocytes, eosinophils, epidermal Langerhans cells	
8. Histamine	Mastocytes (primarily, serosal)	Immunohistochemistry Histochemistry	Some neurons, histamine-containing epithelial endocrine cells of the stomach, basophilic leukocytes, neutrophils, monocytes, macrophages, dendritic cells	
9. Serotonin	Mastocytes (primarily, serosal)	Immunohistochemistry Histochemistry	Neurons, gustatory receptor cells, glomus cells of carotid bodies, enterochromaffin cells, trophoblast, decidual cells, keratinocytes, fibroblasts, melanocytes, platelets	
10. Vascular endothelial growth factor receptor-1 (VEGFR-1)	Mastocytes	Immunohistochemistry	Endothelial vascular cells, epithelial cells of the uterus and bronchi, type 2 pneumocytes, cytotrophoblasts, interstitial cells of testicles, Т lymphocytes, monocytes, micro- and astroglia, neurons	Also used as a tumor marker

Note: the most effective markers for mast cell detection exhibiting the highest specificity are highlighted in bold; numbers 6–10 represent less selective mast cell markers, their usage can result in obtaining erroneous results.

Some methods used for human mast cells visualization can also be successfully applied to detect mast cells of laboratory animals that is necessary to develop adequate biological models of the diseases proceeding under an inflammatory response. Unfortunately, the most specific techniques based on immunohistochemical reactions to tryptase and chymase of human mast cells cannot be used to detect mast cells in animals. It is due to both: the difference in enzyme set of mast cells in different mammals and human, and also a low homology degree of the genes encoding the proteins under study that results in the necessity to use different sets of species-specific antibodies.

Correct fixation of the biological material is of great importance for histochemical and immunohistochemical detection of mast cells. Mastocytes, especially mucosal, are better stained by aniline dyes after using Carnoy’s fixative and the fixatives containing such metal salts as lead and zinc. Short fixation of the material in formalin is sufficient for immunohistochemical mast cell detection, although the best results can be obtained when using zinc-containing fixative solutions.

The most significant technological approach is the use of fluorescent immunohistochemistry techniques and a multimarker analysis combined with confocal microscopy. One of the remaining challenges of mast cell imaging is the lack of standardized approaches for their reliable detection in different animal species, so it can bring in uncertainty when studying the findings of experimental investigations.
